# Supplementary material for: MetaRibo-Seq measures translation in microbiomes
Source: Nat Commun. 2020 Jun 29;11:3268. doi: 10.1038/s41467-020-17081-z (PMC7324362; doi:10.1038/s41467-020-17081-z)
Supplement: Supplementary file 10 — Supplementary Data 7 [file 41467_2020_17081_MOESM10_ESM.zip › File2/Confidence_High_Taxonomy/12123_out.krona.html]

Javascript must be enabled to view this page.

members
magnitude
magnitudeUnassigned
count
unassigned
taxon
rank

12123\_out

10

2
superkingdom
8

phylum
1239
8

186801
class
8

8
order
186802

family
541000
8

8
216851
genus

8

SRS023971\_contig\_number\_contig-100\_5574.5574SRS100021\_contig\_number\_11653SRS1041091\_contig\_number\_7405SRS105153\_contig\_number\_35326SRS1055043\_contig\_number\_20049SRS1055099\_contig\_number\_contig-100\_4493.4494SRS144506\_contig\_number\_contig-100\_5192.286478SRS147022\_contig\_number\_contig-100\_8756.70755
853
species

2

SRS058070\_contig\_number\_contig-100\_30260.30260SRS142890\_contig\_number\_contig-100\_30756.30756
